# C-Reactive Protein as a Therapeutic Target in Age-Related Macular Degeneration

**DOI:** 10.3389/fimmu.2018.00808

**Published:** 2018-04-19

**Authors:** Blanca Molins, Sara Romero-Vázquez, Pablo Fuentes-Prior, Alfredo Adan, Andrew D. Dick

**Affiliations:** ^1^Institut d’Investigacions Biomèdiques Agustí Pi i Sunyer (IDIBAPS), Hospital Clínic de Barcelona, Barcelona, Spain; ^2^Molecular Bases of Disease, Biomedical Research Institute Sant Pau (IIB Sant Pau), Barcelona, Spain; ^3^Universitat Autònoma de Barcelona, Bellaterra, Spain; ^4^Academic Unit of Ophthalmology, School of Clinical Sciences, University of Bristol, Bristol, United Kingdom; ^5^Academic Unit of Ophthalmology, School of Cellular and Molecular Medicine, University of Bristol, Bristol, United Kingdom; ^6^National Institute for Health Research (NIHR) Biomedical Research Centre at Moorfields Eye Hospital, University College London Institute of Ophthalmology, London, United Kingdom

**Keywords:** C-reactive protein, macular degeneration, aging, inflammation, retina

## Abstract

Age-related macular degeneration (AMD), a retinal degenerative disease, is the leading cause of central vision loss among the elderly population in developed countries and an increasing global burden. The major risk is aging, compounded by other environmental factors and association with genetic variants for risk of progression. Although the etiology of AMD is not yet clearly understood, several pathogenic pathways have been proposed, including dysfunction of the retinal pigment epithelium, inflammation, and oxidative stress. The identification of AMD susceptibility genes encoding complement factors and the presence of complement and other inflammatory mediators in drusen, the hallmark deposits of AMD, support the concept that local inflammation and immune-mediated processes play a key role in AMD pathogenesis that may be accelerated through systemic immune activation. In this regard, increased levels of circulating C-reactive protein (CRP) have been associated with higher risk of AMD. Besides being a risk marker for AMD, CRP may also play a role in the progression of the disease as it has been identified in drusen, and we have recently found that its monomeric form (mCRP) induces blood retinal barrier disruption *in vitro*. In this review, we will address recent evidence that links CRP and AMD pathogenesis, which may open new therapeutic opportunities to prevent the progression of AMD.

## Introduction

Age-related macular degeneration (AMD) is the primary cause of irreversible vision loss among the aging population worldwide. The disease affects up to 1.75 million individuals alone in the United States, and this number could increase up to 3 million by 2020 (1–3). Worldwide, the projected number of people with AMD in 2020 is 196 million (95% CrI 140–261), which increases to 288 million in 2040 (205–399) (4). AMD is a complex, degenerative, and progressive disease involving multiple genetic and environmental factors, which can ultimately result in severe visual loss. The disease-causing molecular mechanisms remain unknown, although inflammatory processes have been implicated by the identification of AMD susceptibility genes encoding complement factors (5, 6) and by the presence of complement proteins in drusen, the hallmark deposits associated with AMD and other features of immune activation, including inflammasome activation (7–10).

The pathology of AMD is characterized by vision loss due to alterations in the macula, the central zone of the retina. Visual dysfunction in AMD is associated with the degeneration of the outer portion of the retina, the outer blood retinal barrier (oBRB), which includes the retinal pigment epithelium (RPE), the Bruch’s membrane, and the choriocapillaris. This is followed (or in some cases preceded) by degeneration of the light-sensing photoreceptor cells supported by the oBRB. Degeneration of the RPE seems to begin with impaired clearance of cellular waste. The initial clinical manifestations of AMD are characterized by the presence of drusen, deposits of extracellular matrix, and pigment that form most commonly within the macula at the choroid–RPE interface. Based on the size and number of drusen, the presence of atrophy, and/or neovascularization, AMD is classified into five stages of increasing severity (11). Early and intermediate AMD are characterized by the presence of small or large drusen and RPE irregularities. Forms of late AMD include geographic atrophy and neovascularization, both of which can lead to severe central visual impairment and legal blindness due to degenerative and neovascular alterations in the macula, respectively (11, 12). Although, currently, neovascular AMD can be controlled with antiangiogenic agents that block vascular endothelial growth factor, most treated patients still suffer from visual impairment as they develop fibrosis and atrophy, and more than one-third of them show long-term loss of effect (13). Most concerning is that there is still no approved treatment for geographic atrophy.

Age is the primary risk factor for AMD. Physiological changes that occur with aging may impair cellular function in those at risk of the disease (14). In addition, other genetic and environmental risk factors are associated with AMD, most significantly smoking (15). A variety of complement pathway-associated gene variants, such as complement factor H (CFH) (16), factor B, and the complement components C2 and C3 have associations with AMD pathogenesis (17). Smoking increases the risk of the exudative type of AMD both in females and men (18, 19), and there is a direct association between AMD and raised concentration of cholesterol (20). In addition, small increases in the plasma levels of C-reactive protein (CRP) are an additional associated risk factor for AMD (21). Dietary interventions with carotenoids, oral supplementation with high levels of antioxidants and minerals, or high intake of omega-3 fatty acids and fish arguably slow the course of the disease and are implemented clinically to various degrees worldwide (22, 23). Light and photosensitization reactions may also play a role in the development of AMD *via* synthesis of reactive oxygen species, with consequent damage to the RPE and Bruch’s membrane (24). Finally, chronic systemic disorders such as atherosclerosis (25), diabetes (26), and cardiovascular diseases (27) contribute to the risk for AMD development.

Although the etiology of AMD in terms of multifactorial risk factors are increasingly well documented, the patho-etiology of how oxidative stress, atherosclerotic-like changes, RPE cell dysfunction, genetic variants, and inflammation/altered tissue immune responses interlink is less well defined (28–30). One notion to enquire further is the influence of systemic immunity or alarming, acute phase responses in the progression of AMD, not dissimilar to dementia (31). In this context, elevated CRP levels are found both in the blood of AMD patients and in the eyes of carriers of a *CFH* polymorphism associated to the risk for developing the disease, providing a molecular clue to AMD pathogenesis and to how genetic risk factors may influence its course (21, 32). In this review, we summarize the main findings that support the implication of CRP in the pathogenesis of AMD and its connection with aging.

## Inflammation and AMD

Chronic inflammation is a prolonged condition in which tissue injury and attempts at repair coexist, leading to tissue remodeling and dysfunction. It is the common pathological basis for age-associated diseases such as cardiovascular disease, diabetes, cancer, Alzheimer’s disease, but also AMD. A multitude of bodily changes occur with aging that contribute to the initiation and development of inflammation. In particular, the immune system of elderly individuals is characterized by a basal systemic inflammatory state, as increased levels of proinflammatory cytokines and acute phase reactants are observed with aging (33). Local inflammation and immune-mediated processes play a central role in AMD pathogenesis (34–36).

A competent immune system in the eye is necessary to maintain intraocular health. The network of macrophages and microglia along with the RPE and choroidal endothelial cells maintain tissue homeostasis allowing cellular debris removal and pathogen surveillance. Besides the presence of tissue-resident immune cells, inflammatory molecules are constitutively expressed in the subretinal space, meaning that there is a persistent inflammatory state, known as para-inflammation, which deals with danger signals and protects the tissue against over-inflammation and destruction. Proteomic and histochemical analysis of ocular drusen have shown that these deposits contain inflammatory proteins and complement components that mediate local inflammation, such as C5, C9, CRP, amyloid A, fibrinogen, and vitronectin (7, 37, 38). The complement system is one of the main effectors of the innate immune response. The activation of the complement system culminates in the formation of the membrane attack complex and, potentially, cell lysis. Accumulation of membrane attack complex in the macula increases with aging and in AMD patients compared to age-matched controls (39–42). On the other hand, some diseases that are associated with complement activation have been independently linked to AMD (43), and a number of complement pathway-associated genes have been recognized as important driving factors of AMD pathogenesis. Some of these genetic variants might cause the complement system to be overactive, resulting in a chronic inflammatory condition (42, 44). This abnormal inflammatory stimulus adversely affects RPE cells and promotes drusen formation (45). The strongest genetic risk factor for AMD known to date is a common polymorphism in the *C*FH gene (c.1277T > C, p.Tyr402His); the CFH p.Tyr402His variant (in following termed CFH_H402_) increases the risk for AMD approximately twofold to fourfold for heterozygous and fivefold to sevenfold for homozygous individuals (5, 16, 46).

FH is a major inhibitor of the alternate complement pathway that regulates complement activation in plasma, host cells, and tissue, in particular, at sites of tissue inflammation, following injury or during degeneration (47). The protein is essentially comprised of 20 tandem Sushi domains, also known as short consensus repeat (SCR) or complement control protein modules. The exchanged residue in the FH_H402_ variant is located in domain SCR7, which mediates the binding to CRP, malondialdehyde (MDA), and to cell surfaces through interactions with heparan sulfate (HS) chains (48, 49). The “at risk” variant of FH shows an impaired binding to these ligands, which could result in increased complement activation and chronic local inflammation. MDA is a toxic by-product of lipid peroxidation and Weismann et al. showed that FH binds MDA through SCR7 and protects from oxidative stress. Notably, the “at risk” variant resulted in severely reduced factor-I-mediated C3 cleavage when bound to MDA (50). Regarding HS, the “non-risk” variant of FH can bind to multiple sites on HS chains in BM due to its wide specificity. Instead, the 402H variant only binds to highly sulphated motifs within HS (51, 52). Thus, if insufficient FH is present in BM, as is the case for the 402H variant, there will be increased activation of the complement cascade and the release of pro-inflammatory mediators. However, increased inflammation could be also due to the impaired binding of CRP to the FH from the “at risk” variant.

## Structure and Function of CRP

CRP is the prototypical acute-phase reactant and an active regulator of the innate immune system; CRP levels increase rapidly in response to infection, inflammation, and tissue injury (53). It is a highly conserved protein of the pentraxin family, mainly produced in the liver. Among the multiple functions ascribed to CRP are activation of the classical complement pathway and inactivation of the alternative pathway (53). In plasma, CRP exists as a cyclic, noncovalent pentamer of 125 kDa composed of five identical subunits (pCRP), and which is stabilized by numerous electrostatic and Van-der-Waals interactions (54, 55). Native pCRP binds in a Ca^2+^-dependent manner to phosphocholine (PCh)-containing ligands such as pneumococcal cell wall C-polysaccharide, but also to the surface of necrotic cells and parasites (55–58). Oxidative stress, low pH, and bioactive lipids from activated or damaged cells can dissociate the CRP pentamer into its 23-kDa subunits (59–62). This poorly soluble, tissue-based monomeric form (mCRP) possesses distinct biological functions compared to pCRP (60, 63–67). The dissociation mechanism of CRP requires, first, a reversible structural transition within pCRP subunits, but without disrupting the pentameric symmetry (60, 68, 69). This rapid conversion to the modified form (pCRP*) may contribute to acute phase amplification of the inflammatory response. Then, the pCRP* → mCRP irreversible transition is likely to occur at sites of persistent chronic inflammation, where the inflammatory microenvironment—characterized by acidic conditions, oxidative stress, and presence of bioactive phospholipids—continuously favors dissociation of the pentameric arrangement. mCRP would then effectively trigger proinflammatory responses and regulate complement (68). Indeed, the dissociation of circulating pCRP to mCRP in areas of inflammation has been observed *in vivo* in a rat model of acute inflammation. Mechanistically, this process is dependent on exposure of lysophosphatidylcholine (LysoPCh), a bioactive lipid that is generated after phospholipase A2 activation on activated cell membranes (62, 70).

**Figure 1:**
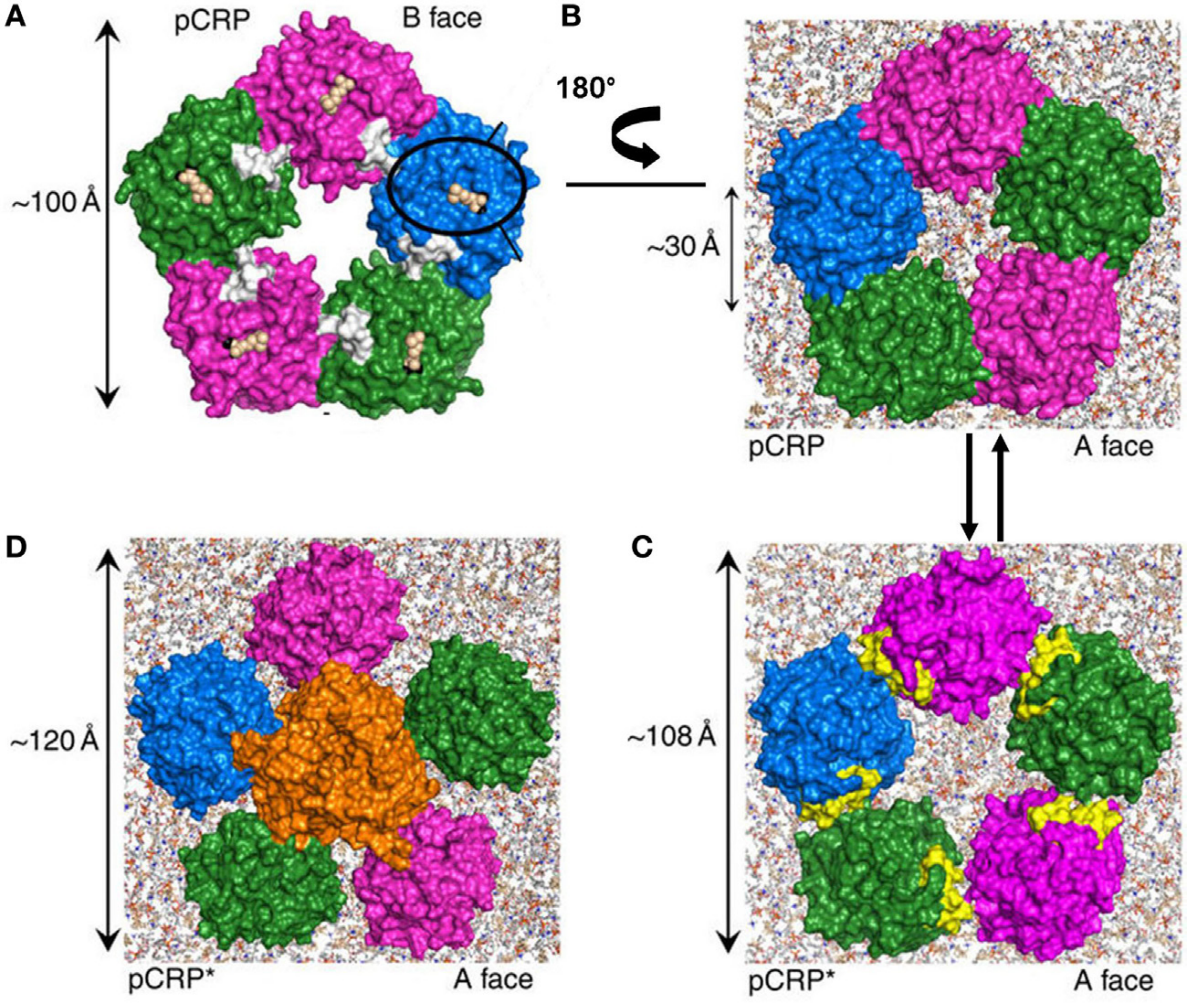
Proposed model of the conversion from the strained to the relaxed conformations of pentameric C-reactive protein (CRP). **(A)** Solid surface representation of the crystal structure of human CRP in its “strained” conformation (pCRP) bound to phosphocholine (PCh) (PDB entry 1B09) (54). The view shown is from the membrane binding “B face” of pCRP. The individual subunits are represented color-coded, with PCh (cream spheres) and Ca^2+^ ions (black spheres) occupying the ligand binding site on each subunit. **(B)** Modeled interaction of pCRP with a phospholipid bilayer. View is from “above,” looking down onto the pCRP “A face.” Each pCRP subunit can independently bind to a PCh head group of the bilayer. Exposure to lysoPCh triggers reversible conversion of pCRP to pCRP*. **(C)** Pentameric pCRP*, same view as in **(B)**. As the individual CRP subunits move apart, a neoepitope (colored yellow) is exposed. **(D)** The globular head of C1q inserts itself into the inner annular void of pCRP* forcing the subunits further apart [adapted from Braig et al. (68)].

The crystal structure of native pCRP in complex with PCh shows how large PCh-containing ligands may be specifically bound by CRP and offers clues to the mechanism of mCRP formation (54). The PCh ligand-binding site is located in a groove of a β-sheet on the so-called “B face” of the pentamer (Figure 1). Multipoint attachment of this planar face of the CRP molecule to a PCh-bearing surface would leave available, on the opposite A face, the recognition sites for complement C1q. In the absence of Ca^2+^, residues 140–150 form a loop that projects away from the body of each CRP subunit exposing a normally hidden proteolysis site. Cleavage at this site facilitates that individual CRP subunits move apart, thus exposing a neoepitope (residues 199–206, colored yellow in Figure 1C) that is recognized by anti-mCRP-specific antibodies (9C9 or 3H12) (68, 71). The globular head of C1q is then able to insert itself into the inner annular void of pCRP* (the relaxed conformation) forcing the subunits further apart (noteworthy, C1q is unable to bind to the “strained” pCRP conformation) (68). Finally, the CRP subunits might dissociate, likely accompanied by partial unfolding to generate the mCRP form (72, 73). This process would enable CRP to target physiologically and/or pathologically significant complement activation.

## Molecular Changes in the OBRB in AMD: Interaction Between CRP and FH

The fact that patients with AMD and individuals with the CFH_H402_ variant show increased systemic and local levels of CRP, respectively, provides a molecular hint on the pathogenesis of AMD. AMD lesion formation has been proposed to share mechanisms with atherosclerotic plaque formation, which is initiated with low-density lipoprotein retention within the arterial wall (74). Although a thorough discussion of the cross-connections between CRP and cardiovascular diseases is beyond the scope of this review, it is noteworthy that patients with clinical evidence of atherosclerosis (stroke, coronary, and peripheral artery disease) show modestly but significantly increased CRP levels (25, 75, 76).

Seddon and coworkers were the first to address the relationship between elevated CRP concentrations and AMD progression. They found a significant increase in circulating CRP levels as the disease progressed, and showed that low-, medium-, and high-risk AMD groups are associated with serum CRP concentrations below 0.5, between 0.5 and 10.0, and over 10.0 mg/L, respectively (21, 77, 78). However, this association has not been universally confirmed (79). A more recent study by the Seddon group shows that high levels of circulating CRP are associated with a higher risk of AMD, regardless of the *CFH* genotype (80).

Other authors have recently attempted to triangulate the association between plasma concentrations of CRP, four *CRP* genetic variants reported to influence CRP circulating levels, and the risk of advanced AMD (81). They found that two of the genetic variants do share some association with plasma CRP concentrations. However, none of the four variants was significantly associated with the risk of AMD. Their findings have important implications for our understanding of the pathophysiology of AMD, in particular, for the distinct roles played by local and systemic inflammation in this regard. However, it must be considered that other factors such as *C3* genotype and smoking strongly affect circulating CRP levels. Thus, these results do not preclude a direct link between AMD pathophysiology through complement activation and chronic inflammation, and plasma CRP concentrations might still be useful as an AMD biomarker. Important differences exist between systemic inflammation and the local inflammatory macular tissue microenvironment.

Since CFH risk haplotypes are associated with increased complement activation in human macular tissue (82) but not in the circulation (83, 84), it is important to determine the localization and abundance of both CRP and FH in the extra-macular choroid of individuals homozygous for the high-risk CFH_H402_ genotype, as compared to those homozygous for the low-risk CFH_Y402_ variant. This investigation, reported by Johnson and colleagues, showed that the localization and abundance of FH do not differ between *CFH* genotypes (32). However, choroidal immuno-staining of CRP was significantly higher in the CFH_H402_ eyes compared to the CFH_Y402_ eyes. Interestingly, these differences between the *CFH* homozygotes were independent of AMD status. Because the high-risk allele affects binding of FH to CRP (85), deficient FH binding could potentially increase the pro-inflammatory activity of CRP in choroidal tissue, contributing to AMD pathogenesis. Also along these lines, Bhutto and colleagues have reported distinct patterns of localization for FH and CRP in the aging eye. Most notably, these authors found an inverse relationship between CRP and FH levels in macular tissue from patients with advanced AMD as compared to age-matched control individuals (86). In AMD patients, Bruch’s membrane, drusen, and choroidal vessel walls all showed increased labeling of CRP and decreased labeling of FH compared to controls. These findings support the idea that the macula of AMD patients has an increased inflammatory microenvironment with decreased capacity for complement inhibition.

**Figure 2:**
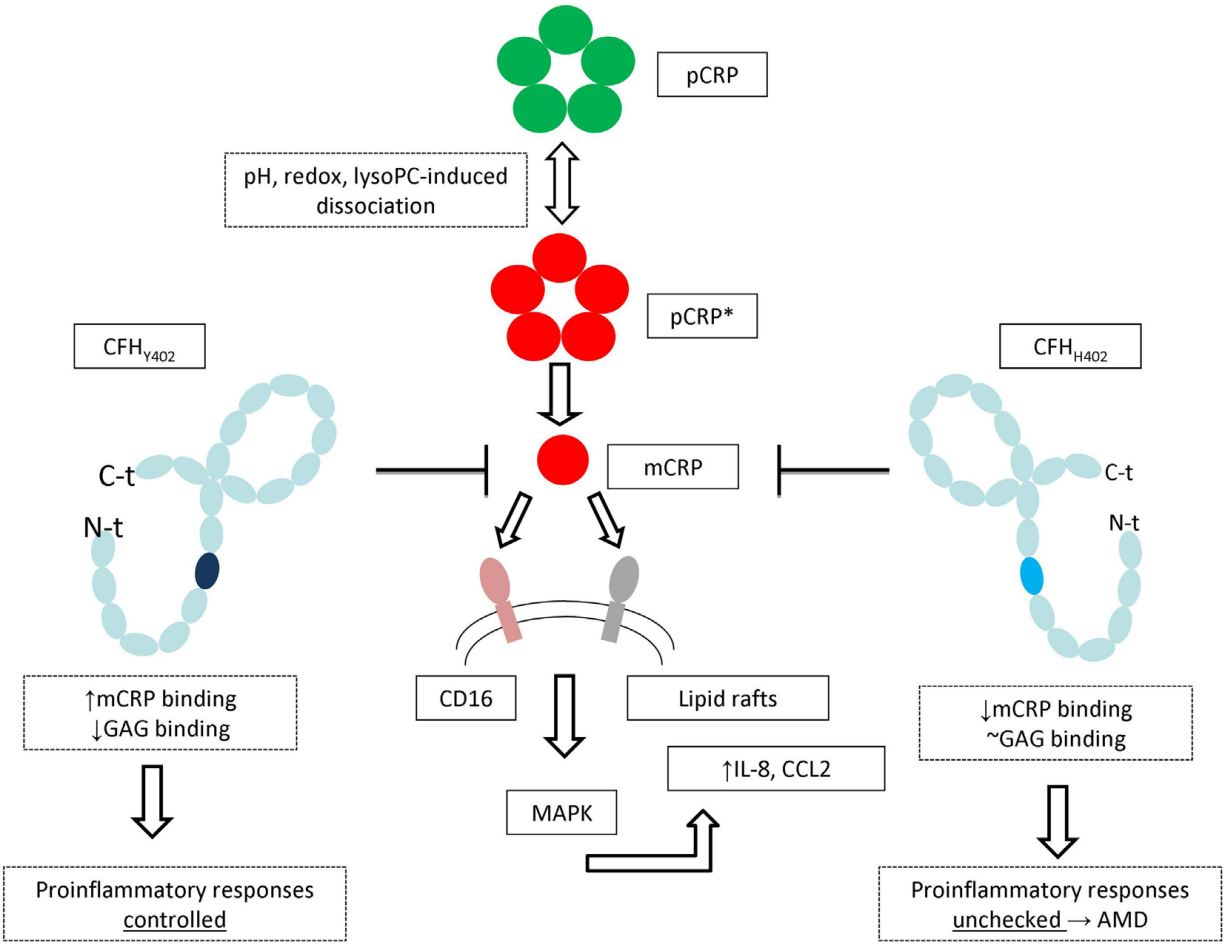
A unified mechanism of mCRP-induced proinflammatory responses and the role of the *CFH* p.Tyr402His polymorphism in age-related macular degeneration (AMD). Generation of mCRP is accelerated *in vivo* under inflammatory conditions by bioactive lipids such as lysophosphatidylcholine (lysoPC) exposed on the surface of microparticles, activated or damaged cells. mCRP is recognized on the cell surface, leading to activation of MAPK pathways and ultimately enhances expression of proinflammatory cytokines and disrupts the outer blood retinal barrier. Binding of FH to mCRP attenuates this inflammatory response, but the FH_H402_ variant is less effective in this regard, both because of its altered binding to glycosaminoglycans (52), but, in particular, due to its markedly lower affinity for mCRP. The unchecked inflammatory response leads eventually to progression of AMD and vision loss [figure adapted from Molins et al. (90)].

Although FH is known to bind CRP, there was certain controversy regarding the relevance of the monomeric and pentameric forms in this regard. For instance, two separate binding sites for pCRP were located on domains SCR4-6 and SCR16-20, respectively (49). On the other hand, FH showed strong binding to denatured, monomeric CRP, rather than to the native multimeric form (87, 88). We have recently shown that mCRP, but not the pentameric form, contributes to oBRB disruption *in vitro* (89). The functional integrity of the RPE, critical for the maintenance of the specialized environment of the neural retina, is dependent on the structures of tight junctions. Exposure to mCRP, but not pCRP, significantly increased the paracellular permeability of the RPE compared with that of untreated cells, suggesting that mCRP could compromise the barrier function of the RPE monolayer. Notably, mCRP was also able to disturb the expression and distribution of the TJ proteins, ZO-1, and occludin. In another study, we also showed that mCRP confers a proinflammatory phenotype to RPE cells as it increases production of the proinflammatory cytokines IL-8 and CCL2 (Figure 2) (90). The mCRP-induced pro-inflammatory phenotype was further demonstrated by the significantly increased rates of peripheral blood mononuclear cells migration treated with conditioned medium from RPE cells after being exposed to mCRP, but not with conditioned media from either untreated cells or from cells exposed to pCRP. The oBRB disruption induced by mCRP could conceivably permit passage of inflammatory cells into the retina, further contributing to chronic inflammation and accelerating tissue damage.

Moreover, we also showed that the “non-risk” FH variant (CFH_Y402_) can effectively bind to mCRP to dampen mCRP proinflammatory activity. Notably, FH from AMD patients carrying the risk polymorphism for AMD shows an impaired binding to mCRP and, therefore, its proinflammatory effects remain unrestrained (Figure 2) (90). In line with and highlighting our findings, Chirco et al. have recently shown that mCRP is the more abundant form of CRP in human RPE-choroid, and that mCRP levels are elevated in individuals with the high-risk CFH geno-type (91), which could thus sustain chronic inflammation contributing to the progression of AMD in CFH_H402_ individuals. Moreover, pro-inflammatory mCRP significantly affects endothelial cell phenotypes, suggesting a role for mCRP in choroidal vascular dysfunction in AMD as well.

It is also interesting to note that, in our cohort of AMD patients, those carrying the risk variant of *CFH* had significantly higher levels of systemic IL-8 and CRP than healthy subjects carrying the non-risk allele (90). Further, the levels of these proteins were positively correlated in AMD patients homozygous for the risk *CFH_H402_* variant. These results are in conflict with previous studies showing that CRP levels and the *CFH_H402_* polymorphism were independent risk factors for AMD (80). We observed differences in circulating CRP concentrations between subjects carrying the different *CFH* variants, albeit in a smaller population. Our results might explain the previously reported higher risk of AMD within genetically susceptible individuals when CRP concentrations are high (80). We hypothesize that higher levels of circulating CRP could derive in higher mCRP concentrations in microenvironments that favor dissociation, such as inflammatory or apoptotic conditions, which in the case of patients carrying the *CFH_H402_* risk variant would further cause unchecked inflammation. However, it is unclear where, when, and how mCRP dissociates within the oBRB. mCRP could either dissociate distantly in activated endothelium or locally within the RPE. Unchecked mCRP activity may sustain chronic inflammation thus favoring AMD progression. Whether this provokes disease or not requires validation, but this process alone may not be sufficient to explain all the immune-related changes we observe in AMD and, therefore, further research is warranted.

## Targeting Monomeric CRP in AMD

The recent findings from us and others discussed above reinforce the importance of mCRP in chronic inflammation and point to the pCRP dissociation process and/or mCRP itself as novel therapeutic targets for AMD. Indeed, therapies associated with a reduction in systemic CRP levels are successfully used in other chronic inflammatory diseases such as atherosclerosis, where CRP is an important player (76). However, given that CRP may have a more important role in the macular tissue, it might be more appropriate to target local CRP for AMD treatment. Blocking the dissociation of pCRP with 1,6-bis-PCh, a compound that stabilizes CRP in a decameric form, abolished the proinflammatory effects of mCRP *in vivo* (62). Restrictively, 1,6-bis PCh is not suitable for clinical purposes due to its pharmacokinetics and its low affinity for pCRP (150 nM) (62). Thus, a more potent drug with higher oral bioavailability, higher affinity for pCRP, and prolonged half-time needs to be designed to efficiently target the pCRP dissociation process as an innovative therapeutic strategy. Blocking LysoPCh formation with PLA2 inhibitors may be another interesting approach to inhibit pCRP dissociation. Alternatively, therapeutic approaches aimed to enhance FH-mCRP binding could be developed to block mCRP proinflammatory activities, thus preventing the progression of AMD.

## Conclusion

The reduced ability to control the balance between pro- and anti-inflammatory signals associated with aging might promote a switch to chronic inflammation in the macular tissue. This scenario could then favor CRP dissociation and mCRP accumulation further fueling chronic inflammation and tissue damage, especially in those patients with the “risk” FH variant, CFH_H402_, where FH is unable to dampen mCRP proinflammatory activity and to localize to HS in Bruch’s membrane. A combination of poor binding of the FH H402 variant to Bruch’s membrane and mCRP, combined with aging associated processes such HS loss and an increased proinflammatory environment, may eventually result in complement activation, persistent mCRP-induced inflammation, and thereby contribute to AMD progression. Future research is warranted to confirm the contribution of mCRP to disease etiology and progression, and eventually to test the therapeutic potential of compounds that either prevent CRP dissociation or stimulate FH binding to mCRP.

## Author Contributions

BM, PF-P, SR-V, AA, and AD contributed to the design of the project and manuscript preparation, and all authors reviewed the manuscript.

## Conflict of Interest Statement

The authors declare that the research was conducted in the absence of any commercial or financial relationships that could be construed as a potential conflict of interest.
